# Thoughts and Experiences of Behçet Disease From Participants on a Reddit Subforum: Qualitative Online Community Analysis

**DOI:** 10.2196/49380

**Published:** 2023-12-12

**Authors:** Jenny Xiaoyu Li, Elaine Yacyshyn

**Affiliations:** 1 Department of Medicine University of British Columbia Vancouver, BC Canada; 2 Division of Rheumatology Department of Medicine University of Alberta Edmonton, AB Canada

**Keywords:** Bechet disease, Behçet, online community, Reddit, vasculitis, quality of life, QoL, qualitative, community, morbidity, support, diagnosis, symptoms, vascular, vascular system, vascular disease

## Abstract

**Background:**

Behçet disease (BD) is a type of vasculitis with relapsing episodes and multisystemic clinical features, associated with significant morbidity and impact on patients’ lives. People affected by BD often participate in discussions of their illness experiences. In-person support groups have limited physical accessibility and a relative lack of anonymity; however, online communities have become increasingly popular.

**Objective:**

This study investigates the perspectives and experiences of people affected by BD by examining the content shared and discussed on a subforum of the website Reddit—a popular online space for anonymous discussions.

**Methods:**

All discussion threads posted between March 9, 2021, and March 12, 2022, including posts and comments, were examined from the subforum “r/Behcets,” an anonymous online community of 1100 members as of March 2022. A Grounded Theory analysis was completed to identify themes and subthemes, and notable quotes were extracted from the threads. Parameters extracted from each post included the number of comments, net upvotes, category, and subcategories. Two research team members read the posts separately to identify initial codes and themes to ensure data saturation was achieved.

**Results:**

Six recurring themes were identified: (1) finding connectedness and perspectives through shared experiences, (2) struggles of the diagnostic odyssey, (3) sharing or inquiring about symptoms, (4) expressing strong emotions relating to the experience of BD, (5) the impact of BD on quality of life and personal relationships, as well as (6) COVID-19 and the COVID-19 vaccination in relation to BD. Subthemes within each theme were also identified and explored.

**Conclusions:**

This novel study provides a qualitative exploration of the perspectives and experiences of people affected by BD, shared in the anonymous and accessible online community of Reddit. The study found that people impacted by an illness seek to connect and receive validation through shared conditions and experiences. By examining the content shared in r/Behcets, this study highlights the needs of people affected by BD, identifying gaps and areas for improvement in the in-person support they receive.

## Introduction

Behçet disease (BD) is a type of vasculitis with relapsing episodes and multisystemic clinical features. Symptoms include oral ulcers as well as a range of manifestations that can involve the articular, neurologic, ocular, vascular, gastrointestinal, urogenital, pulmonary, and cardiac systems [1,2]. Although rare, BD affects people from around the world, and it is relatively more common among patients from the Silk Road demographic (including regions in the Middle East and Asia) [1,3]. Specifically, the prevalence of BD in the United States is 5.2 per 100,000 population, while prevalence has been reported to be as high as 420 per 100,000 in Turkey [3,4].

BD is associated with significant morbidity and disease burden, with a more severe disease course in the young adult population [1]. For individuals who are affected by rare diseases like BD, it can be challenging to find others who have the same condition to discuss shared experiences. Online networks are conveniently accessible [5] and free from the constraints of space, travel, and scheduling [6]. These online communities also provide a means for people to obtain both support and information [6]. Many of those affected by rare diseases view the connections made online as critical in providing support and validation for their experiences [7,8].

Reddit is a public online space that allows users to anonymously share content, thoughts, insights, information, and feelings on a wide variety of topics. It is the seventh most frequently visited website in the United States and 20th in the world, with more than 52 million daily active users, 100,000 online communities, and 50 billion monthly views [9,10]. Users can publish content by starting a “thread,” beginning with a “post” under which other users can write comments and have discussions. Reddit contains subforums called “subreddits,” each of which is a community with members who post, comment, and have discussions on a specific topic. Content published on the Reddit platform regarding medical conditions has been the subject of a number of qualitative studies in recent years [11-13].

Although there are local or national groups supporting people affected by BD, online communities allow people to connect without the limitations associated with the rarity of the disease within a particular geographical area [14,15]. r/Behcets is an online community that is dedicated to the discussion of BD. It is a “subreddit” (ie, a subforum of Reddit) and was created in 2015, with around 1100 members as of March 2022. The anonymity of subreddits as online communities makes them a particularly revealing and valuable source of information regarding the thoughts and emotionally engaged perspectives of internet users on the topics of discussion. This is especially apparent when compared to platforms like Facebook, which displays the names and identities of the users [16,17]. Additionally, unlike some online platforms like Twitter, Reddit does not have a limitation on the word or character count of each post, allowing users to express themselves more freely. Although readers cannot confirm the diagnostic status of online community members, the posts are reflective of perspectives from those who may still be in the diagnostic process (ie, suspected, confirmed, or in the process of ruling out BD)—similar to many patients in the real world.

This paper presents a qualitative study of the perspectives and experiences of people affected by BD. Our overall aim was to explore the content shared and discussed on a subforum of the website Reddit—an online space for anonymous discussions—and to identify themes reflecting critical areas of patient concerns.

## Methods

### Procedure

In this qualitative analysis, a grounded theory design was selected to analyze Reddit threads. Threads from the subreddit r/Behcets were arranged by their original date of posting. All threads started from March 9, 2021, to March 12, 2022, were sequentially examined, including both posts and comments. Through a qualitative exploration based on principles of Grounded Theory [18], overarching themes were identified from the posts and comments, and subthemes were found within each theme. Representative quotes were selected. Two team members (JXL and EY) conducted initial coding and theme identification from the Reddit threads. Independent analyses were completed by the two authors, and consensus was reached. Furthermore, parameters were extracted from each post, including the date of the original post, number of comments, net upvotes, poster-selected tag, category, subcategories, type of poster, and status of diagnosis. These parameters are defined and described in Table 1. Qualitative analysis of data was completed once saturation was reached.

**Table 1:** Explanation of parameters.

Parameter	Explanation
Date of the original post	The date when the original post was published on Reddit.
Number of comments	The number of comments responding to the post or to other comments.
Net upvotes	The number of total upvotes minus downvotes from Reddit users.
Poster-selected tag	The tag phrase, known on Reddit as a “flair,” that a Redditor may attach to their post. The options for the r/Behcets subforum are the following: general question, diagnosis help, symptoms, treatments, research or study, and patient support or story.
Category	The category of the post as determined by the authors of this study (eg, patient support)
Subcategory	The specific topic of the topic within its respective category (eg, patient support—experiences and quality of life)
Type of poster	Whether the writer of the post is a person with diagnosed or suspected Behçet disease or someone posting for or with regards to a person with diagnosed or possible Behçet disease.
Status of diagnosis	Whether the writer of the post or person discussed in the post has been already diagnosed with Behçet disease.

### Ethical Considerations

The project was submitted to the University of Alberta Research Ethics Board for review, which determined the project did not require ethics approval due to the use of publicly available data. In addition, every effort was taken to remove patient or user identifiers and maintain the anonymity of the posts; if users choose to remove their posts from Reddit’s public platform, the data in this paper could not be traced back to their usernames or identities.

## Results

### Overview and Themes

A total of 196 threads were examined; their characteristics are summarized in Tables 2-4. In total, 6 themes and 16 subthemes were identified. Analyses were continued until data saturation occurred. Themes, subthemes, and quotes are described in the following sections and in Table S1 in Multimedia Appendix 1.

**Table 2:** Summary of posts extracted. The date range of original posts was from March 9, 2021, to March 12, 2022 (N=196).

Characteristics		Values
Comments per post, range		0-57
Comments per post, mean		8.0
Net upvotes per post, range		1-40
Net upvotes per post, mean		7.7
**Poster types, n (%)**		
	Patient	171 (87)
	Friend or family	12 (6)
	Unknown	13 (7)
**Status of diagnosis, n (%)**		
	Diagnosed	43 (22)
	Implied	93 (48)
	Suspected	46 (23)
	Unknown	14 (7)
Age of patients^a^, mean		25

^a^If mentioned by the original poster.

**Table 3:** Number of threads with each post-selected tag.

Post-selected tag	Threads, n
Symptoms	36
General question	61
Treatments	35
Diagnosis help	29
Research or study	6
Patient support or story	25
None	4

**Table 4:** Number of threads in each category and subcategory of topics.

Categories and subcategories		Threads, n
**Symptoms (n=46)**		
	Oral ulcers	4
	Genital ulcers	2
	Other dermatological symptoms	3
	Joint pain	1
	Neurologic symptoms	9
	Gastrointestinal symptoms	1
	Ophthalmological symptoms	2
	Other symptoms	9
	Symptom patterns	17
**Patient support (n=38)**		
	Communities	5
	Experiences and quality of life	33
**General topics (n=36)**		
	Curiosity and speculation	7
	Requesting information	6
	Sharing information	7
	COVID-19 and vaccination	14
**Treatments (n=37)**		
	Options	5
	Treatment-specific inquiry	19
	Nonpharmacological treatments	3
	Barriers to treatment	4
	Other treatment-related inquiries	2
	Treatment side effects	4
**Diagnosis (n=34)**		
	Seeking advice	31
	Investigations	3
Miscellaneous		5

### Theme 1: Finding Connectedness Through Shared Experiences

#### Subtheme 1.1: Feeling Understood by Others Affected by This Rare Condition

It was expressed that “it’s very difficult to explain this disease and the severity of it to anyone since it’s so rare.” One member wrote that “for years, [they] honestly felt like the only person on the planet with Behcet’s because [they had] never met anyone else with the disease.” One user specifically stated that they “need advice from people who live with this [condition].” Another poster, after a chain of responses to their post, wrote the following:

Thank you so much again for all the helpful advice. Behcet’s is so uncommon, so this [subforum] continues to be so helpful.

#### Subtheme 1.2: Inquiries and Discussions Regarding Similarities in Symptoms

BD can present with a constellation of symptoms that vary from person to person, and many posters wondered if other people share the same symptoms they have, asking questions such as “Has this happened to anyone else?” or “Is it just me?”

#### Subtheme 1.3: Seeking Perspectives From Others Who Have Tried a Treatment Option

A total of 19 posters specifically inquired about the thoughts and experiences of others regarding specific medications or disease management options. For example, after a poster asked, “Is it normal to have these types of side effects” to medication, other community members commented, describing their personal experiences with the medication, to which the poster responded, “Thank you so much, this is super helpful.”

### Theme 2: The Struggles of the Diagnostic Odyssey

#### Subtheme 2.1: The Lengthy Diagnostic Odyssey

The struggles of the diagnostic odyssey are commonly discussed in the community. One patient described having seen “18 doctors of different specialties” over the course of 1 year and being given “different types of medication to try and treat symptoms before they knew” before reaching the diagnosis of BD. Another poster described their diagnostic odyssey as “years of ruling things out.” It is common for users to share stories like having “had symptoms for years and [having] been to like half-a-dozen doctors before finding one who knew what was wrong.” There were also complaints regarding the lack of knowledge from health care professionals, as one user described going to “a rheumatologist who barely even knew what Behcet’s was.” The process can be so difficult that one user shared the following comment: “I really thought that the only way we would ever get an answer was going to be through an autopsy.”

Diagnosis can be particularly difficult to reach for those who do not fit into the “Silk Road” demographic. One user who described themselves as having some Russian and Scottish heritage shared that they “kept getting told [that there was] no way [they] have Behcet’s” until they mentioned “Turkey” in their blood. In response, a commenter shared that “[their] first rheumatologist was convinced” that they could not have BD “without Middle Eastern ancestry.”

#### Subtheme 2.2: Negative Experiences With Physicians and the Health Care System

Posters described feeling dismissed, such as one user who “felt unheard and unacknowledged.” In response, a comment expressed similar experiences with this rare disease, as follows:

I know it’s frustrating. I’ve had my fair share of meeting new medical professionals and they haven’t even heard of Behcet’s.

Anxiety in seeing physicians was also expressed, as the following comment, in which a user requested advice on interacting with her physician:

How do I [bring] this up with my doctor? I don’t want her to think Google is smarter than her, but I am concerned that since this is so rare, she wouldn’t look into it…I’ve had pretty terrible doctors in the past, and I think I do have anxiety about doctors in general.

There were also those who felt heard by their physicians who “didn’t dismiss [them].” For example, one user shared this gratitude toward physicians who take the time to listen:

Thankfully, I did find a doctor [whom] I’ve actually stuck with…he spent nearly an hour writing up my symptoms and genuinely seemed to want to get to the bottom of this.

#### Subtheme 2.3: Presenting to Health Care Providers Outside of Rheumatology

For some users, the first suspicion of BD did not come from rheumatologists. Even a poster who had a rheumatologist shared that it was their dentist who recognized that the patient’s BD symptoms warranted another rheumatology consult. Gynecology is another setting to which people with BD often present. One user reported that a gynecologist who examined their ulcers “immediately said ‘Behcet’s’.” However, genital ulcers associated with BD are not always so easily recognized. One user wrote, “I was diagnosed with herpes more times than I can count.” Eventually, it was a dermatologist who saw this patient and expressed strong suspicions for BD. The misinterpretation of ulcers as sexually transmitted infections (STIs) can be stressful and embarrassing, “like going through humiliating experience after humiliating experience to get diagnosed.”

### Theme 3: Sharing or Inquiring About Symptoms

#### Subtheme 3.1: Stress as a Major Trigger

When discussing stress, community members appear to have a shared understanding of the significance of stress as “a major trigger,” with a commenter who wrote the following:

Another user commented about stress, and I will double on that. Stress is able to shoot up flares on its own, so keep it down.

Likewise, another user shared that “Stressing out about the physical symptoms compounds them tremendously.”

#### Subtheme 3.2: The Severity of Symptoms

For patients with BD, symptoms can feel “just uncomfortable and awful.” A user with “extreme leg and arm pain” described that they would “cry because [the extremities] hurt so bad.” A patient with genital ulcers described “crying from the pain” during urination or having to wear a dress without underwear “because everything hurt” when wearing underwear with pants. However, the severity and impact of the symptoms are not necessarily apparent to others when “often times, the reaction is ‘but you don’t look sick.’”

#### Subtheme 3.3: The Large Variety of Symptoms

BD presents with a large range of symptoms affecting different body symptoms. The thread titled “What are your symptoms?” had 57 comments, which was the largest number of comments among the threads examined in this study. There was a remarkable variety of symptom combinations among the community members, ranging from the commonly discussed (eg, oral ulcers) to less commonly mentioned (eg, blood clots). As one user concluded, “It is so different for everyone.”

### Theme 4: Expressing Strong Emotions Relating to the Experience of BD

#### Subtheme 4.1: Feeling Lonely and Misunderstood

Misunderstandings about this rare condition can contribute to the emotional distress experienced by people with BD. An example was that “many people with Behcet’s get misdiagnosed as HSV-2 [herpes simplex virus type 2],” which can be “extremely damaging, embarrassing, and even downright traumatizing.” One user from a cultural context where virginity was “very important” described being put “through a virginity test” because she was mistakenly diagnosed with an STI, which was “horrible” for her.

Loneliness was another recurring emotion that was cited as motivation for members to join this online community. One poster described feeling “so utterly alone.” This condition is also described as “the loneliest experience,” as it can be challenging to feel validated or understood in in-person communities.

#### Subtheme 4.2: Mental Health and Emotions

The impact of BD on mental health is frequently discussed. Users described “struggling with depression and anxiety in such an immense way,” having “bursts of tears and stress throughout the day,” “exhaustion at never being able to do anything right,” and feeling “incredibly depressed and hopeless.”

One user wrote that it feels “scary” when receiving this diagnosis while having the “worst pain of your life.” Another member described feeling “gross” and “betrayed” in their body. The snowball effect of negative emotions is also demonstrated by another frustrated poster who wrote the following comment:

I just feel like my world is falling apart right now…I feel worthless and it’s out of my control. I’m just in a really dark place. I truly do not know how to recover from this.

### Theme 5: The Impact of BD on Quality of Life and Personal Relationships

#### Subtheme 5.1: Impact on Activities and Endeavors

The impact of BD on hobbies, interests, and personal activities is frequently discussed in r/Behcets. For example, one user wrote, “I fear beyond words…I am only 19 and it messes with so much.” Users expressed feeling “so frustrated” about aspects such as “the loss of mobility” and “the inability to have a regular exercise schedule.”

Trying to make peace with the limitations on their life, a user stated that they do their “absolute best to not let [Behçet disease] affect [their] life,” but the fatigue and soreness during their flare-ups would cause them to put their goals and projects “on the back burner for a few days,” illustrating the disruptive effect of the disease on people’s lives.

#### Subtheme 5.2: Impact on Education and Work

BD can cause significant disruptions in education and professional capacity, especially during the years of growth for young adults. A student wrote, “I feel like I can’t keep up with school with all the days I need to rest….” Similarly, a working member shared, “I feel like I’m not valuable to workplaces because of all the random time I require off when I get sick….”

#### Subtheme 5.3: Impact on Personal Relationships

Intimate relationships can be impacted by symptoms of BD, especially ulcers or symptoms in the oral and genital regions. One user who had “a hard time even picturing being with someone these days” described feeling as if they had an STI, “despite not having been sexually active in a long time.” Another frustration was that “kissing hurts.” The impact of the disease on relationships is further illustrated by someone who posted the following:

It has ruined my relationship with my boyfriend…I feel sick at the thought of being touched right now, and I’m hurting him.

### Theme 6: COVID-19 and COVID-19 Vaccination in Relation to BD

Many posts and comments examined in this study were from a time that was significantly impacted by the COVID-19 pandemic.

#### Subtheme 6.1: Inquiring About COVID-19 and Vaccinations

Posters asked other community members about the impact of COVID-19 and the vaccinations in relation to their BD and medications. For example, one user was “curious to hear how [the vaccination] has been for fellow Behcets/Remicade patients,” and another wondered about “any reactions to the COVID vaccine while on Humira.”

#### Subtheme 6.2: Sharing Experiences With Vaccination

Users shared their experiences with the COVID-19 vaccines with other community members. One user wrote, “I’m just excited to get it all out of the way…I can try my best to answer any questions.” Seeing one another’s experiences provided “another data point to show the vaccine is safe for our small patient community.”

## Discussion

### Principal Findings

This study examines the perspectives and experiences of people affected by BD based on the content shared on the subforum r/Behcets, which provides a valuable source of data due to its anonymity, user-guided discussions, and abundance of active discussions without the restrictions of geographical proximity. By developing an understanding of themes and aspects of care that are important from the perspectives of patients, care providers can provide more patient-centered care and develop stronger therapeutic relationships with their patients.

Many users remain active in this online community, which demonstrates the unique value of these anonymous discussions among those who are impacted by the condition. This shows that people affected by the illness seek not only information but also an outlet and validation for their disease experiences. As such, this study further validates the theory that validation of disease experiences is important in the care of patients with rheumatic diseases [19].

The users in the subforum start discussions with topics or questions of their choosing, and other users are free to respond in the manner and timing of their choosing. This environment differs from physical support groups by enabling individuals to collectively make sense of their shared condition at their own pace. It also allows them to select discussions to respond to while avoiding the pressure to engage in topics in which they do not wish to participate. Although in-person groups encourage real-time dialogue, online participants have more time to formulate and articulate their thoughts in written form.

Studies have shown the significant impact of BD on the quality of life and life satisfaction of patients [20-25]. Patients’ quality of life and perceptions of wellness are becoming increasingly acknowledged as end points and targeted outcomes in studies [26]. The illness perception of patients can also play a significant role in the relationship between symptom severity and perceived pain or energy level [27]. This study is unique in its examination of anonymous, user-initiated, and narrative discussions to further understand areas that are prioritized by people impacted by BD.

Experiences with misdiagnosis are common, not only in failing to differentiate BD from other rheumatologic diseases but also in the lack of suspicion for a rheumatologic etiology behind the patient’s presentation. Diagnosis can be challenging to reach, with the clinical presentation ranging from classic findings to atypical or incomplete presentations [28,29]. Many users with BD report first being suspected of this disease by health care providers from outside rheumatology, such as gynecologists or dentists. This raises the importance of generalists and nonrheumatology specialists, to whom patients often present earlier with their symptoms, in recognizing the possibility of this rare rheumatological disease as well as making timely referrals to rheumatologists or other relevant specialists.

Although the relative lack of physician expertise in BD can play a role in the experiences of patients, users who expressed frustrations with physicians usually focused more on the negative experiences of feeling dismissed or unheard rather than the knowledge base of the physician. Patients who expressed gratitude for their physicians often focused on the physician’s willingness to understand rather than their medical expertise.

BD can have a significant impact on quality of life and psychological well-being [21,30-32]. Intimate relationships are also negatively affected by BD [33]. This was demonstrated in the discussions in r/Behcets, where users often described the challenges in romantic relationships while having symptoms that affect the oral and genital regions. The genital symptoms in BD are often misattributed to STIs, which can cause significant psychological trauma. Therefore, mental health and psychological well-being should be considered when caring for patients with BD.

By examining this online content, it appears to be commonly felt that there is a lack of understanding of both the severity and range of symptoms attributed to BD. It is important to have an appreciation for the disease experience, not only for physicians but also for other individuals who have an impact on the patients’ lives, such as romantic partners, teachers, and employers, all of whom can play a role in patients’ diagnostic process and quality of life.

With symptoms that often peak in early adulthood, BD can greatly impact growth, productivity, and personal endeavors. This further illustrates not only the significant impact of BD on livelihood but also the importance of receiving diagnosis and treatment in a timely manner to preserve the quality of life of patients with BD.

Future research is needed to further identify and characterize the barriers preventing patients with BD from obtaining timely and optimal care, improve diagnostic practices to shorten the diagnostic odyssey, implement strategies to improve patient satisfaction, and provide means of emotional support that are preferred by patients.

### Limitations

We could not ensure that everyone who posts in this online community was formally diagnosed with BD. However, those who posted likely have some resemblance to patients in clinical practice who may be at various points of the diagnostic process (ie, suspected, confirmed, or in the process of ruling out BD). Next, the forum is an English-language space, which may limit the contributing user base to people who know English and choose to use it for online discussions. The prevalence and presentation of BD can differ with varying patient demographics [2], and the members of this English-speaking online community do not necessarily represent the global BD community. Furthermore, another study has found that individuals who seek health-related information and support on the internet are generally younger and possibly more affluent than those who seek this information through in-person means [5,34]; therefore, patients who do not seek help in this specific online setting are not represented in this study. Another limitation of this study is that the r/Behcets subforum is moderated by a human individual. Posts that are deemed inappropriate for the online community can be removed by the moderator of the subreddit and are not publicly accessible.

### Conclusions

This novel study provides a qualitative exploration of the perspectives and experiences of people affected by BD, which were shared in the anonymous and accessible online community of Reddit. The study found that people impacted by an illness seek to connect and receive validation through shared conditions and experiences. Overarching themes that emerged include the following: the challenges in the diagnostic process; the severity and variety of symptoms; the impact on numerous aspects of one’s quality of life; and the need to express emotions, such as frustration, fear, and shame. This understanding can encourage more empathetic care by shining light on the needs of people affected by BD, gaps and areas for improvement in the in-person support received by those impacted by the disease, and the need for adequate awareness of the disease from a wide spectrum of care providers.

## Appendix

Multimedia Appendix 1 Overarching themes, subthemes, and corresponding quotes in the examined Reddit threads.

## Abbreviations

BD: Behçet disease
HSV-2: herpes simplex virus type 2
STI: sexually transmitted infection

## Data Availability

Data that support the findings of this study are available from the r/Behcets subforum of Reddit and publicly accessible [35].
