# Supplementary material for: Thoughts and Experiences of Behçet Disease From Participants on a Reddit Subforum: Qualitative Online Community Analysis
Source: JMIR Form Res. 2023 Dec 12;7:e49380. doi: 10.2196/49380 (PMC10751625; doi:10.2196/49380)
Supplement: Multimedia Appendix 1 [file formative_v7i1e49380_app1.docx]

**Appendix**

*Table S1. Overarching Themes, Subthemes, and Corresponding Quotes*

| **Overarching Themes** | **Subthemes** | **Corresponding Quotes*** |
| --- | --- | --- |
| 1. Finding connectedness and perspectives through shared experiences | 1.1. Feeling understood by others affected by this rare condition | - *“Can anyone talk with me about this who can relate?”* - *“I feel your pain… But I got through it. And so will you.”* |
|  | 1.2. Inquiries and discussions regarding similarities in symptoms | - *“Does anyone smell smoke?”* - *“Elbows, wrists and fingers aches, anyone?”* |
|  | 1.3. Seeking perspectives from others who have tried a treatment option | - *“Has anyone experienced relief from pain and eye symptoms from Otezla?”* |
| 2. The struggles of the diagnostic odyssey | 2.1. The lengthy diagnostic odyssey | - *I never go a day without pain and was misdiagnosed time and time again...”* - *“I was just diagnosed after ~13 years of being dismissed and misdiagnosed”* |
|  | 2.2. Negative experiences with physicians and the healthcare system | - *“I’ve dealt with so many doctors who just completely brushed everything under the rug and [acted] like you’re not critically ill [so] they can’t do anything!”* |
|  | 2.3. Presenting to healthcare providers outside of rheumatology | - *“I know a couple of people with Behcet’s who had no luck with rheumatologists but have with other specialists...”* - *“My dentist is the one who realized something was off and told me to consult my rheumatologist.”* |
| 3. Sharing or inquiring about symptoms | 3.1. Stress as a major trigger | - *“Stress is a very real trigger.”* - *“Stress is a huge deal in Behcet’s. It triggers all the symptoms and makes them almost unbearable. Do whatever is reasonable to reduce stress in your life. That helps the most.”* |
|  | 3.2. The severity of symptoms | - *“Most people do not understand how bad the pain actually is.”* - *“I remember crying from the pain every time I had to pee.”* |
|  | 3.3. The large variety of symptoms | - *“My symptoms are not all ‘usual’, which I found out later is actually common amongst Bechet’s.”* - *“Consistent mid day nausea off and on for the last year. Have you experienced this?”* - *“I just thought [the tremor] was from a medicine I was taking, but I stopped the medicine… and I still have them… just one more thing to add to the list of symptoms.”* |
| 4. Expressing strong emotions relating to the experience of Behcet’s disease | 4.1. Feeling lonely and misunderstood | - *“So few people out there understand this particular kind of misery.”* - *“I feel so alone in my hospital bed... I am so frightened.”* |
|  | 4.2. Mental health and emotions | - *“I feel incredibly depressed and hopeless.”* - *“Though I've been more-or-less stable and healthy [mentally] for years, I experienced crippling anxiety and suicidal depression - much of it directly related to Behcet's for over a decade."* |
| 5. The impact of Behcet’s disease on quality of life and personal relationships | 5.1. Impact on activities and endeavors | - *“Running is one of my hobbies but it's almost impossible for me to enjoy now...”* - *“My last [tattoo] was so bad! Crazy swelling and redness that never happened when I was younger and healthier… It was awful.”* |
|  | 5.2. Impact on education and work | - *“I have yet to recover from the first flare up and now this happens. I was just starting to get my life back, got a job I love, got an offer to coach a junior high boys basketball team, and then this happens… lost it all.”* - *“I can relate. I'm 41 now and doing well, but Behcet's kicked my ass all throughout my teens and early twenties. I almost flunked out of college because I was so fatigued and in pain all the time. It was embarrassing.* |
|  | 5.3. Impact on personal relationships | - *“How do they feel about kissing, knowing all the chaos in my mouth?”* - *“I’m not embarrassed of having an autoimmune disease, but I am very self conscious when it comes to explaining the details of it to a new partner.”* |
| 6. COVID-19 and/or the COVID-19 vaccination in relation to Behcet’s disease | 6.1. Inquiring about COVID-19 and vaccinations | - *“I'm curious what to expect from getting my booster vaccine in a few weeks. Any stories? Take care everyone”* |
|  | 6.2. Sharing experiences with vaccination | - *“Reading people's COVID vaccine experience posts was super helpful [thank you!], so I thought I'd leave one as well.”* |

* More corresponding quotes are described under their respective themes and subthemes.
